# Global Routine Vaccination Coverage — 2012

**Published:** 2013-11-01

**Authors:** Dept of Immunization, Vaccines, and Biologicals, World Health Organization, Geneva, Switzerland, United Nations Children’s Fund (UNICEF), New York, New York, Global Immunization Div, Center for Global Health, CDC

In 1974, the World Health Organization (WHO) established the Expanded Programme on Immunization to ensure that all children have access to routinely recommended vaccines (1).* Despite improvement in global coverage with the third dose of diphtheria-tetanus-pertussis (DTP) vaccine (DTP3), from 5% in 1974 to 83% in 2011, almost one fifth of the world’s children still had not received their third dose of the DTP series during their first year of life. In May 2012, the World Health Assembly endorsed the Global Vaccine Action Plan (GVAP) to guide the Decade of Vaccines’ vision to extend benefits of immunization to all persons. GVAP’s key indicators include achieving and sustaining 90% national DTP3 coverage and ≥80% DTP3 coverage in every district by 2015. During 2012, as in the 2 preceding years, an estimated 83% of infants worldwide received 3 doses of DTP vaccine; however, coverage varied among the WHO regions. Among 194 WHO member states, 131 (68%) achieved ≥90% DTP3 national coverage, and 59 (30%) achieved ≥80% DTP3 coverage in every district. However, 22.6 million children did not receive 3 DTP doses, a key indicator of immunization program performance. Strengthening national immunization systems, especially in countries with the greatest number of undervaccinated children, should be a global priority to reduce morbidity and mortality from vaccine-preventable diseases.

Vaccination coverage is calculated as the percentage of persons in the target age group who received a vaccine dose by a given age. Administrative coverage can estimate vaccination coverage as the number of doses of a specific vaccine dose administered through routine immunization services to those in the target age group divided by the estimated target population. DTP3 coverage by age 12 months is a major indicator of immunization program performance; coverage with other vaccines, such as a third dose of polio vaccine (Polio3) or first dose of measles-containing vaccine (MCV1) are also assessed. Countries report administrative coverage annually to WHO and UNICEF (2). Immunization coverage surveys also can be used to estimate vaccination coverage. A representative sample of households is visited to identify children in the target age group. Dates of vaccination are transcribed from the child’s vaccination card or recorded based on caregiver recall. WHO and UNICEF derive national coverage estimates through an annual country-by-country review of all available data, including administrative and survey-based coverage; as new data are incorporated, revisions of past coverage estimates (3) and updates are published on their websites.† This report is based on these WHO and UNICEF estimates of vaccination coverage.

Estimated global DTP3 coverage among infants aged <12 months in 2012 was 83%, ranging from 72% in the WHO African Region to 97% in the Western Pacific Region, and representing 110.6 million vaccinated children (Table). Estimated global coverage with bacille Calmette-Guérin (BCG) vaccine, Polio3, and MCV1 were 89%, 84%, and 84%, respectively. During 2012, 131 (68%) countries achieved ≥90% national DTP3 coverage, and 59 (30%) achieved ≥80% DTP3 coverage in every district. DTP3 coverage was 80%–89% in 34 (18%) countries, 70%–79% in 13 (7%) countries, and <70% in 16 (8%) countries.

Among the 22.6 million children who did not receive three DTP doses during the first year of life, 16.3 million (72%) lived in 10 countries, among which 12.4 million (55%) lived in three countries: 30% in India (72% DTP3 coverage), 17% in Nigeria (41% DTP3 coverage), and 7% in Indonesia (64% DTP3 coverage) (Figure). An estimated 12.6 million (56%) children did not receive the first DTP dose, while nearly 10 million (44%) started but did not complete the 3-dose series.

Vaccines are increasingly being introduced into national immunization programs. By the end of 2012, hepatitis B vaccine was included in routine childhood vaccination schedules in 181 (93%) countries; 94 (52%) recommended administering the first dose within 24 hours of birth to prevent perinatal hepatitis B virus transmission. Worldwide, coverage with 3 doses of hepatitis B vaccine (including countries that have not introduced the vaccine) was 79%, ranging from 72% in the WHO South-East Asia Region and African Region to 91% in the Western Pacific Region (Table). Coverage with 3 doses of *Haemophilus influenzae* type b vaccine, which had been introduced into 184 (91%) countries by 2012,§ was 45% globally,¶ ranging from 11% in the South-East Asian Region to 91% in the Region of the Americas. By 2012, rotavirus vaccine was introduced in 41 (21%) countries, and pneumococcal conjugate vaccine (PCV) in 88 (45%) countries. Coverage with the completed rotavirus vaccination series (2 or 3 doses, depending on vaccine used) was 11% globally, but reached 69% in the Americas. Coverage with 3 doses of PCV was 19% globally and was highest (77%) in the Americas. A second dose of MCV (MCV2) is recommended in 146 (75%) countries; however, because of difficulties with aggregating and compiling reported data on MCV2 coverage, WHO and UNICEF do not estimate global MCV2 coverage.

## Editorial Note

In 2012, approximately 110 million infants (83%) worldwide received ≥3 doses of DTP vaccine, an indicator of overall vaccination coverage; however, approximately 22.6 million children did not receive 3 doses, leaving them susceptible to vaccine-preventable diseases and death. More than half of incompletely vaccinated children live in only three countries, underscoring the need to strengthen routine immunization systems in countries with the highest number of incompletely vaccinated children.

In 2010, the global health community launched the Decade of Vaccines Collaboration, with the vision of extending benefits of immunization to all persons. GVAP outlines steps to achieve this vision and includes an accountability framework requiring annual reporting of immunization indicators to the World Health Assembly. Although two thirds of countries achieved the GVAP target of 90% national DTP3 coverage, fewer than one third achieved >80% DTP3 coverage in every district, highlighting the need to reduce disparities in coverage within countries.

Administrative coverage estimates are convenient and timely, but they might overestimate or underestimate coverage if inaccuracies occur in the numerator (i.e., doses administered) or denominator (i.e., census data). In contrast, coverage surveys are not dependent on knowing target population size, nor are they subject to some limitations of administrative data sources (e.g., dependency on denominator data); however, they are costly and do not provide timely information to guide programs. In addition, coverage survey results for multidose antigens are increasingly subject to bias as vaccination card retention rates decline and reliance on maternal recall for more vaccines and multiple doses increases (4).

Vaccination coverage estimates in this report are based on doses provided to infants aged <12 months. GVAP’s emphasis on equity of vaccination services across the life span, including children aged >12 months, means that the need for coverage estimates with vaccines offered after age 1 year will increase. Ascertaining coverage with the second dose of measles vaccine (MCV2) will become more important as measles elimination efforts continue, especially with increasing use of the MCV2 visit as a platform for delivery of other health services and vaccinations. Among countries where MCV2 is routinely recommended, 40% offer it during the second or third year of life, 54% at ages 3–7 years, and 6% at an age >7 years. In countries with high rates of measles transmission, MCV2 is recommended at age 15–18 months. This variability in the age of vaccination will create challenges in aggregating and compiling country data into global coverage estimates. Challenges immunization programs face in monitoring administrative MCV2 coverage estimates include difficulty monitoring vaccination coverage among children aged >1 year, the potential for misclassification of an MCV1 dose in a child age >1 year as an MCV2 dose, the misclassification of campaign doses as routine MCV1 or MCV2 doses, and inaccuracies in population estimates in older age groups. MCV2 coverage surveys are complicated by the low rate of vaccination card retention among parents of older children.

What is already known on this topic?

Substantial progress has been made in reducing vaccine-preventable morbidity and mortality since establishment of the global Expanded Programme on Immunization in 1974. However, millions of children, especially those in less developed countries, still are not being reached by the program.

What is added by this report?

During 2012, estimated global coverage with the third dose of diphtheria-tetanus-pertussis vaccine (DTP) was 83%. India, Nigeria, and Indonesia accounted for 55% of the 22.6 million children who had not received 3 doses of DTP by age 1 year. Worldwide coverage with other recommended vaccines was 89% for bacille Calmette-Guérin vaccine, 84% for the third dose of poliovirus vaccine, 84% for the first dose of measles-containing vaccine, 79% for the third dose of hepatitis B vaccine, and 45% for the third dose of *Haemophilus influenzae* type b vaccine. Among all incompletely vaccinated children, 56% had never received the first dose of DTP vaccine.

What are the implications for public health practice?

Many children, especially those in less developed countries, remain at risk for vaccine-preventable diseases. Strategies to improve vaccination coverage might differ for those children who have never been vaccinated, compared with those who have started but not completed the immunization series.

Among all incompletely vaccinated children worldwide, nearly 10 million received ≥1 DTP dose, but failed to complete the 3-dose series; however, 12.6 million (56%) never received the first DTP dose. Factors associated with undervaccination might differ from those associated with nonvaccination. For example, immunization system weaknesses (e.g., inadequate vaccine supply, poor health worker availability and knowledge, and insufficient political and financial support) are more commonly associated with undervaccination, whereas parental attitudes and knowledge about immunization appear to play a greater role among children who have not started vaccination (5). To achieve improvements in vaccination coverage globally, multifaceted and country-specific strategies will be required to address factors contributing to incomplete infant vaccination, especially in countries with the largest numbers of incompletely vaccinated children.

## Figures and Tables

**FIGURE:**
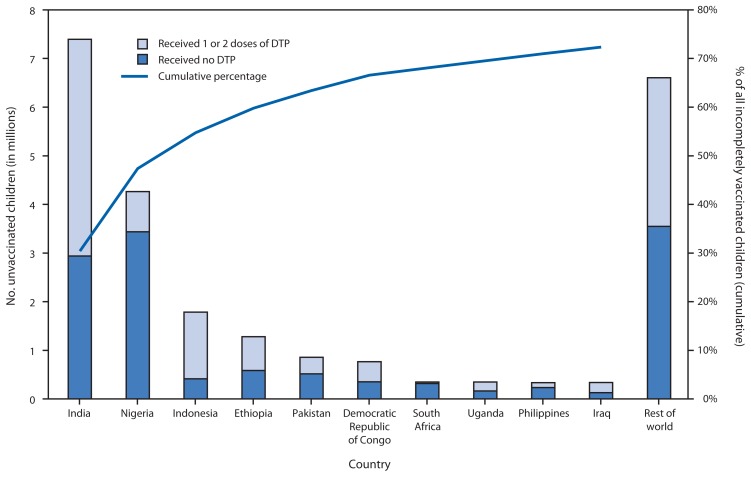
Estimated number of children who had not received 3 doses of diphtheria-tetanus-pertussis vaccine (DTP) during the first year of life among 10 countries with the largest number of children incompletely vaccinated with DTP, by country, and cumulative percentage of all incompletely vaccinated children — worldwide, 2012

**TABLE:** Vaccination coverage, by vaccine and World Health Organization (WHO) region* — worldwide, 2012

	Vaccination coverage (%)							
		
WHO region	BCG	DTP3	Polio3	MCV1	HepB3	Hib3	Rota last	PCV3
**Total (worldwide)**	**89**	**83**	**84**	**84**	**79**	**45**	**11**	**19**
African	82	72	77	73	72	65	5	21
American	96	93	93	94	91	91	69	77
Eastern Mediterranean	88	83	82	83	81	58	14	13
European	93	95	96	94	79	83	2	39
South-East Asian	88	75	74	78	72	11	—	0
Western Pacific	97	97	97	97	91	14	1	1

**Abbreviations:** BCG = bacille Calmette-Guérin vaccine; DTP3 = 3 doses of diphtheria-tetanus-pertussis vaccine; Polio3 = 3 doses of polio vaccine; MCV1 = 1 dose of measles-containing vaccine; HepB3 = 3 doses of hepatitis B vaccine; Hib3 = 3 doses of *Haemophilus influenzae* type b vaccine; Rota last = last dose of 2- or 3-dose rotavirus vaccine series; PCV3 = 3 doses of pneumococcal conjugate vaccine.

* Weighted regional average.

## Footnotes

* Bacille Calmette-Guérin (BCG), diphtheria-tetanus-pertussis (DTP), polio, and measles vaccines.

† Estimates are available at http://www.who.int/entity/immunization_monitoring/data/coverage_estimates_series.xls and http://www.childinfo.org/immunization.html.

§ Includes parts of Belarus, India, Maldives, and Nigeria.

¶ Excludes India, which does not yet report coverage.

## References

[1] Keja K, Chan C, Hayden G, Henderson RH (1988). Expanded programme on immunization. World Health Stat Q.

[2] CDC (2011). Global routine vaccination coverage, 2010. MMWR.

[3] Burton A, Monasch R, Lautenbach B (2009). WHO and UNICEF estimates of national infant immunization coverage: methods and processes. Bull World Health Organ.

[4] Cutts FT, Izurieta HS, Rhoda DA (2013). Measuring coverage in MNCH: design, implementation, and interpretation challenges associated with tracking vaccination coverage using household surveys. PLoS Med.

[5] Rainey J, Watkins M, Ryman T, Sandhu P, Bo A, Banerjee K (2011). Reasons related to non-vaccination and under-vaccination of children in low and middle income countries: findings from a systematic review of the published literature, 1999–2009. Vaccine.

